# Evaluating the Adversarial Robustness and Clinical Safety of Quantized Hierarchical Transformers for Edge-Based Malaria Microscopy

**DOI:** 10.3390/s26092888

**Published:** 2026-05-05

**Authors:** Umar Hasan, Turki G. Alghamdi, Muhammad Ali Nayeem

**Affiliations:** 1 Department of Electrical and Computer Engineering, School of Engineering and Physical Sciences, North South University, Dhaka 1229, Bangladesh; umar.hasan@northsouth.edu; 2 Department of Computer Engineering and Networks, College of Computer and Information Sciences, Jouf University, Sakaka 72388, Saudi Arabia; talghamdi@ju.edu.sa; 3 Department of Computer Engineering, College of Computer, Qassim University, Buraydah 51452, Saudi Arabia

**Keywords:** artificial intelligence, adversarial robustness, edge AI, generative adversarial networks, malaria diagnostics, post-training quantization, swin transformer, vision transformers

## Abstract

Automated mobile microscopy in Internet of Things (IoT) networks is essential for scaling malaria screening in resource-constrained environments. Deploying standard convolutional architectures here introduces severe adversarial vulnerabilities. Post-Training Quantization (PTQ) mitigates hardware constraints by converting floating-point models to 8-bit integers (INT8); however, its impact on clinical safety and security remains unexplored. This study presents an adversarial audit of quantized Vision Transformers for medical edge deployment. We evaluated a Swin-Tiny transformer against ViT-Tiny and MobileNetV3 baselines using a 27,558-image malaria dataset and an out-of-distribution (OOD) White Blood Cell dataset. Our findings redefine the “Quantization Shield” hypothesis. PTQ compresses the Swin model by 3.9× (to 27.89 MB) with a negligible 0.11% accuracy drop, maintaining statistical reliability on OOD tests. However, the hypothesized architectural resilience shatters under white-box Projected Gradient Descent (PGD) attacks. Despite robustness against single-step attacks, both MobileNetV3 and the INT8 Swin-Tiny collapse to 0.00% accuracy under iterative PGD. Conversely, the quantized Swin-Tiny resists black-box transfer attacks from a surrogate, maintaining 81.00% accuracy. We conclude that while quantized Vision Transformers meet mobile sensor constraints, integer quantization provides zero innate defense against targeted iterative perturbations, exposing a critical vulnerability in diagnostic IoT networks.

## 1. Introduction

Automated mobile microscopy, operating as an optical edge sensor within diagnostic Internet of Things (IoT) networks, represents a paradigm shift in global health. It offers scalable, point-of-care malaria screening in resource-limited regions. Deep learning models embedded directly into these portable sensor pipelines can rapidly analyze blood smears, bypassing the need for scarce trained microscopists for every diagnosis [1,2]. Decentralized diagnostics can significantly reduce mortality rates in endemic zones. However, as documented across the broader landscape of edge artificial intelligence [3], deploying highly accurate models is severely constrained by the limited memory capacity, low computational throughput, and tight energy budgets of edge devices. While standard convolutional architectures have historically met these constraints, Vision Transformers (ViTs) have recently emerged as a highly accurate alternative. Yet, their deployment introduces a severe computational bottleneck. Recent benchmarking demonstrates that standard transformer inference on edge devices is up to 41 times slower than traditional convolutional neural networks (CNNs) [4]. To resolve these hardware constraints without sacrificing the representational power of hierarchical transformers, Post-Training Quantization (PTQ) is employed to convert 32-bit floating-point (FP32) models to 8-bit integers (INT8), though its subsequent impact on adversarial security requires rigorous auditing.

In resource-constrained clinical environments, a misdiagnosis due to a compromised medical IoT sensor is a critical patient safety hazard. PTQ has emerged as a standard architectural solution to edge hardware constraints [5]. By converting neural network weights and activations from FP32 to INT8, PTQ drastically reduces the memory footprint and accelerates inference latency without requiring extensive model retraining [6]. While the efficiency gains of PTQ are well-documented in general computer vision, a critical question remains unanswered in the medical domain: how does integer quantization alter the clinical safety and adversarial security of compressed diagnostic sensors? In practical edge deployments, models operate outside tightly controlled laboratory pipelines and are continuously exposed to noisy optics, variable sample preparation quality, unstable connectivity, and potentially untrusted acquisition environments. Under these conditions, efficiency alone is insufficient; diagnostic systems must also preserve decision integrity under both benign perturbations and intentional manipulations. For this reason, security auditing should be treated as a co-equal deployment requirement alongside memory, latency, and energy optimization for medical edge AI.

Currently, lightweight architectures such as MobileNetV3 [7], and hybrid deep learning models [8] are standard choices for edge deployment due to their optimized parameter counts and computational efficiency. However, medical AI is acutely vulnerable to adversarial attacks, in which mathematically crafted, imperceptible input perturbations can forcibly flip a clinical diagnosis from positive to negative [9]. While MobileNet architectures successfully satisfy hardware constraints, their architectural simplicity and continuous convolutional operations render them highly susceptible to such gradient-based attacks [10].

**Figure 1:**
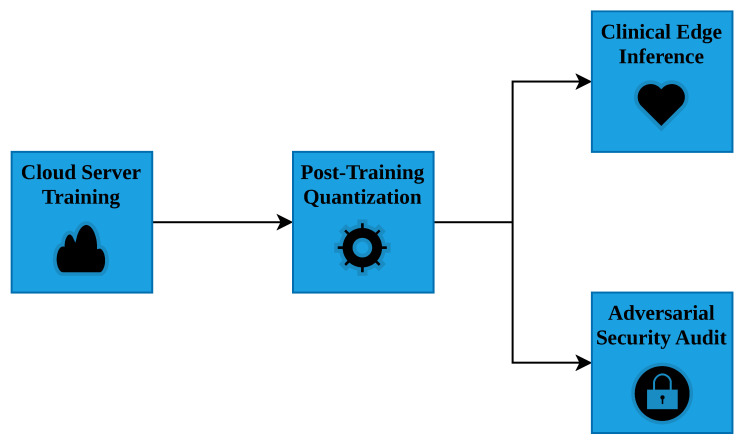
Study Overview. The experimental pipeline illustrates the cloud-based training of the Swin Transformer, its INT8 Post-Training Quantization, and the subsequent dual-track evaluation for clinical edge inference and adversarial security.

In assessing adversarial robustness, it is vital to distinguish between single-step and iterative attacks. Single-step techniques, such as the Fast Gradient Sign Method (FGSM), calculate perturbations based on a single gradient estimation. Iterative techniques, such as Projected Gradient Descent (PGD), repeatedly adjust the perturbation within strict mathematical bounds [11]. Iterative methods act as a much more rigorous evaluation of true model security, as single-step methods can often be defeated by superficial irregularities in the loss landscape known as gradient masking. An overview of our complete experimental workflow is presented in Figure 1.

It is crucial to clarify that this study is fundamentally an investigative security audit and empirical benchmarking analysis, rather than the proposal of a novel defensive architecture or a new neural network paradigm. By systematically combining established PTQ methodologies with advanced hierarchical vision transformers, our objective is to dissect the complex interactions between integer discretization, hardware efficiency, and adversarial vulnerability in practical medical edge deployments.

To the best of our knowledge, this study is the first to combine hierarchical Vision Transformers (ViTs), PTQ, and iterative adversarial robustness in point-of-care medical diagnostics. We investigate the adversarial vulnerabilities of quantized architectures, specifically comparing a 29-million-parameter Swin-Tiny Transformer against a standard ViT-Tiny and MobileNetV3 baseline. This is an investigative benchmarking study focused on empirical auditing and comparative evaluation, rather than the proposal of a new model architecture or defense algorithm. Our core contributions are summarized as follows:

- We evaluate the application of PTQ to the Swin-Tiny model, documenting its effect on the sensor’s memory footprint (a reduction to 27.89 MB), diagnostic accuracy (97.90%), and peak inference RAM utilization (0.01 MB).
- We examine the clinical reliability of the quantized edge sensor compared to the FP32 baseline when exposed to out-of-distribution (OOD) cellular morphologies, utilizing energy-based scoring and McNemar’s statistical test.
- We assess the adversarial robustness of the quantized architecture against single-step (FGSM) attacks, multi-step white-box (PGD) attacks, and black-box transfer attacks. This comprehensively analyzes the impact of discrete INT8 representations on sensor security under targeted and surrogate manipulations, alongside natural sensor noise.

The structure of this article is as follows. Section 2 presents the related work. Section 3 addresses the problem formulation, threat model, and the proposed methods. In Section 4, the performance evaluations are presented. An interpretation of these results is provided in Section 5. Finally, Section 6 offers a summary of key findings, further implications for the field, and our conclusions.

## 2. Related Work

### 2.1. Deep Learning in Malaria Diagnostics

Automated mobile microscopy has become a critical focal point for scaling up malaria diagnostics in endemic regions where laboratory infrastructure is minimal. Recent scoping reviews have highlighted the immense potential of point-of-care technologies to replace manual microscopy [1,2]. Early deep learning approaches predominantly relied on traditional CNNs to automate the detection of Plasmodium parasites in thick and thin blood smears [12]. For example, Yang et al. [13] demonstrated a smartphone-based pipeline utilizing an Iterative Global Minimum Screening technique paired with a custom CNN, achieving 93.46% accuracy. Similar architectures have further improved performance by leveraging end-cloud collaborative frameworks; Cai et al. [14] integrated portable microscopes with modified AlexNet and GoogLeNet models, achieving overall accuracies exceeding 97.7%.

Furthermore, mobile phone-based CNNs have been successfully deployed for specific parasite stage detection in Giemsa-stained smears [15], and fully embedded sample-to-answer devices are currently being prototyped for on-site diagnosis [16]. Beyond standard brightfield microscopy, researchers are also innovating at the optical hardware level. For instance, Holmström et al. [17] demonstrated that deep learning algorithms paired with portable, low-cost fluorescence digital scanners can achieve high precision for point-of-care Plasmodium falciparum detection. Object detection frameworks have also been widely evaluated for use in mobile diagnostics. Nakasi et al. [18] evaluated pre-trained architectures for thick blood smears, and found that Single Shot MultiBox Detector (SSD) architectures were better suited for the computational limits of mobile phone deployment, whereas YOLO-based models also achieved high precision [19].

The deployment of diagnostic models on resource-constrained mobile and Internet of Things (IoT) devices requires balancing computational efficiency with architectural robustness. Object detection frameworks have been widely evaluated for this purpose. A recent study evaluating hybrid deep learning frameworks demonstrated that customized CNNs cascaded with Recurrent Neural Networks (such as LSTMs and BiLSTMs) deployed on point-of-care devices achieve an optimal balance between rapid inference times and acceptable detection accuracy [8]. Furthermore, evaluations of YOLOv5 and Faster R-CNN for automated malaria diagnosis using smartphone microscopes have reported high precision and suitability for low-power hardware [20].

Beyond standard classifications, ensuring anatomical and structural correctness in complex medical imaging has driven the adoption of sophisticated auxiliary modules and structural priors. For instance, recent advancements in cardiac ultrasound segmentation have demonstrated that integrating contour-guided query refinement networks (CGQR-Net) can significantly enhance boundary precision [21]. Similarly, Dense Associative Networks (DANs) have been effectively utilized to enforce input-independent anatomical patterns, thereby improving model robustness under poor visibility conditions [22]. While these methods achieve state-of-the-art structural consistency on unconstrained servers, translating such complex, multi-head architectures to severely constrained mobile point-of-care devices necessitates robust compression techniques.

The broader field of medical image analysis is undergoing a fundamental transition from traditional CNNs to ViTs. ViTs are increasingly integrated into diagnostics because their self-attention mechanisms effectively capture intricate, long-range dependencies in complex medical images [23,24]. To bridge the gap between these heavy transformer architectures and resource-constrained clinical settings, knowledge distillation has proven highly effective. For instance, Hasan and Nayeem [25] successfully utilized Decoupled Knowledge Distillation to transfer diagnostic sensitivity from a heavy Swin Transformer teacher to a lightweight CNN student, drastically reducing computational overhead while maintaining clinical accuracy. Similarly, recent frameworks such as CerebroNet [26] have demonstrated that systematically combining knowledge distillation with unstructured pruning can compress diagnostic models by up to a factor of 17 (requiring merely 0.637 million parameters) while maintaining over 96% accuracy for clinical MRI analysis. To further stabilize transformer training, researchers have addressed the severe class imbalances inherent in malaria datasets. Tan and Liang [27] successfully employed a Wasserstein Generative Adversarial Network with Gradient Penalty (WGAN-GP) to synthesize fake cell images. Despite these successes, ViTs have significant drawbacks on the edge. Zhao et al. [28] noted that while models such as DeiT and Swin achieve superior top-1 accuracy, their significant parameter counts and floating-point operations result in inference latencies up to 25.5 times higher than CNNs on mobile devices. Badar et al. [29] compared a Swin Transformer backbone with a lightweight MobileNet backbone in IoT systems, and found that the lightweight CNN not only satisfied strict parameter budgets but also achieved a higher mean average precision than the transformer. To circumvent the massive computational footprint of Vision Foundation Models in clinical settings, recent approaches like PolySAM-Lite [30] have successfully employed Low-Rank Adaptation (LoRA). By injecting trainable matrices strictly into the attention layers, such frameworks can adapt heavy transformer backbones for medical segmentation while updating less than 5% of the total parameters.

### 2.2. Edge AI and Quantization

The paradigm of edge deep learning integrates computational resources directly at the data source, which is proving to be transformative for real-time medical diagnostics [31]. In the medical domain, this shift is strongly motivated by the need to deliver timely diagnostic support in remote and low-resource settings where cloud access, specialist availability, and stable network infrastructure are often limited. To address the strict hardware limitations of these mobile deployments, PTQ has emerged as a critical model compression technique. A comprehensive scoping review by Paddo et al. [5], alongside recent surveys by He [32], highlighted that techniques such as dynamic pruning and quantization drastically reduce memory footprints across medical imaging applications. Beyond standard single-device quantization, researchers are actively exploring distributed paradigms; for instance, Liu et al. [33] demonstrated that dynamically partitioning deep learning models across resource-constrained IoT devices significantly reduces inference latency for medical analysis. Moreover, hybrid approaches that combine PTQ with QAT are being developed to dynamically adapt to heterogeneous edge constraints [34]. These advanced compression strategies are actively being validated clinically, such as the successful deployment of INT8 quantized architectures for real-time dental diagnostics on custom hardware accelerators [35]. In resource-constrained visual detection tasks, deployment optimization methods such as model compression and quantization are critical for balancing the inference speed with acceptable accuracy [36].

For ViTs, the impact of quantization is substantial but architecturally complex. Liu et al. [6] demonstrated that INT8 quantization consistently reduces ViT model sizes by approximately a factor of four. However, the resulting on-device latency improvements are highly variable; recent benchmarking reveals this is largely due to the inefficient quantization of specific mathematical operators, such as LayerNorm and GELU [4]. This architectural friction is further supported by extensive edge evaluations; for instance, the ETBench suite demonstrated that INT8 quantization significantly underperforms in both accuracy and inference speed compared to FP16 when applied to hybrid ViTs across diverse edge platforms [37]. Similarly, Monteiro et al. [38] empirically proved that while traditional CNNs maintain high accuracy under 8-bit quantization, standard ViTs exhibit significant performance degradation. Furthermore, Lee et al. [39] explained that applying 8-bit integer quantization to hybrid ViTs on mobile devices often leads to a drastic accuracy drop due to highly dynamic ranges and zero-point overflow, complicating their reliable deployment at the edge. Recent advancements have demonstrated the immense hardware benefits of precise 8-bit formats such as Posit8 and FP8, which reduce overall edge accelerator area by 30% to 34% and decrease power consumption by 26% to 32% compared to BFloat16 architectures [40].

### 2.3. The Evolution of Robustness in Medical AI

Comprehensive surveys on medical AI reliability have consistently demonstrated that deep learning models deployed in healthcare settings are highly susceptible to adversarial perturbations, necessitating robust defensive frameworks to ensure patient trust [41]. Building on this, Dietrich et al. [42] highlighted that advanced models used for disease detection and image segmentation in radiology are vulnerable to imperceptible noise, which can cause AI systems to incorrectly classify pathological scans as normal. Dong et al. [43] noted that medical images might be even more susceptible to adversarial attacks than natural images due to their highly standardized nature and overlapping foreground and background features. Li et al. [44] further corroborated this, emphasizing that label scarcity and vastly similar backgrounds in medical scans, such as Optical Coherence Tomography, make them uniquely vulnerable to crafted adversarial noise and necessitate specialized defense frameworks.

This vulnerability extends across various imaging modalities. Haque and Zafar [10] demonstrated that state-of-the-art models analyzing X-ray and CT scans for COVID-19 and pneumonia experience drastic performance degradation when subjected to PGD and FGSM attacks. This catastrophic collapse is a recurring theme; Yinusa and Faezipour [45] recently demonstrated that a medical CNN achieving 98.91% accuracy on clean CT lung scans plummeted to a mere 4.00% under a PGD attack, resulting in complete misclassification of normal cases. Zahoor and Ghani [11] and other recent studies confirm that PGD causes catastrophic degradation in lung X-ray classifications, creating an unacceptable risk of false-positive diagnoses in clinical settings [46]. Similarly, Pal et al. [47] highlighted that both full and partial image perturbations severely compromise the reliability of deep learning diagnostics for skin cancer and Optical Coherence Tomography. The catastrophic consequences of these vulnerabilities are not isolated to healthcare; comprehensive surveys in other safety-critical domains, such as autonomous traffic sign recognition, confirm that deploying even advanced ViTs in adversarial environments remains a critical challenge [48]. Evaluating AI-powered predictive solutions across diverse medical tasks consistently reveals severe degradation under gradient-based attacks [49].

When comparing architectural resilience, ViTs demonstrate far greater natural robustness to standard gradient-based attacks than traditional CNNs [50,51]. However, their reliance on large, attention-driven architectures introduces unique vulnerabilities at the hardware level. For instance, Latibari et al. [52] recently demonstrated that ViTs are highly susceptible to Rowhammer-induced fault injections. Furthermore, hybrid models and distillation pipelines introduce additional risks. Recent findings indicate that transformers trained via knowledge distillation from CNN teachers can inherit the adversarial vulnerabilities of their convolutional counterparts [53]. Xu et al. [54] demonstrated that tokenization strategies heavily influence robustness. By utilizing a linear diffusion equation, scale-space tokenization extracts patches from a higher-dimensional representation, enabling self-attention modules to implicitly learn structural dependencies rather than relying on easily manipulated texture features. However, the intersection of PTQ efficiency and Swin Transformer robustness under active multi-step adversarial conditions remains entirely unexplored.

### 2.4. Emerging Defenses and Ethical Implications

Securing medical AI on edge devices introduces a profound tension between computational cost and clinical safety. To mitigate adversarial threats, researchers are exploring novel defensive architectures. Xu et al. [54] established that scale-space tokenization improves model robustness by extracting image patches from a higher-dimensional representation, enabling ViTs to implicitly learn stronger structural dependencies. Additionally, the integration of Spiking Neural Networks (SNNs) offers a promising defense. Recent work has illustrated that combining sliding-window SNNs with ViTs significantly improves robustness against PGD attacks while maintaining energy efficiency through sparse and asynchronous spiking activity [55]. Drawing inspiration from parallel edge computing sectors, Nassar [56] demonstrated within the Industrial Internet of Things (IIoT) that federated learning combined with SNNs can effectively defend against adversarial poisoning while remaining compact enough for edge deployment. This provides a highly relevant blueprint for securing distributed point-of-care medical networks.

However, these security measures impose ethical and practical trade-offs on the system. Ahadian et al. [57] emphasized that implementing privacy-preserving techniques or robustness mechanisms such as adversarial training often degrades the baseline accuracy of diagnostic models or drastically increases computational overhead. This creates an ethical dilemma in clinical settings, as developers must weigh the necessity of defending against theoretical adversarial threats against the immediate clinical safety requirement of providing highly accurate diagnoses for actual patients.

### 2.5. Summary of Related Literature

To contextualize our contributions within the current landscape of medical edge AI, Table 1 summarizes the most highly relevant literature. The table highlights the prevailing divide in the field: research either optimizes for hardware efficiency (via CNNs and Quantization) or investigates security (via Transformers and adversarial defenses), but rarely bridges the two for mobile clinical deployment.

## 3. Methods

### 3.1. Problem Formulation and Threat Model

The deployment of medical diagnostic models to edge devices can be mathematically formulated as a constrained empirical risk minimization problem. Let $D={\left\{\left(x_{i},y_{i}\right)\right\}}_{i=1}^{N}$ represent the clinical dataset where $x_{i}\in R^{H\times W\times C}$ is a blood smear image and $y_{i}\in \left\{0,1\right\}$ is the corresponding binary label representing parasitized or uninfected states. Our primary objective is to learn a function $f_{\theta }:X\to Y$ parameterized by θ that maximizes the diagnostic accuracy, formulated as:

(1) $$\theta^{*}=\arg\min_{\theta }E_{(x,y)∼D}\left[L\left(f_{\theta }\left(x\right),y\right)\right]$$

where L represents the standard cross-entropy loss function. However, for mobile edge deployment, this optimization is subject to strict hardware bounds:

(2) $$s.t.\;\mathrm{Size}\left(f_{\theta }\right)\leq M_{max},\;\mathrm{MACs}\left(f_{\theta }\right)\leq C_{max}$$

where $M_{max}$ and $C_{max}$ denote the absolute memory footprint and computational throughput limits of the target microcontroller or smartphone processor.

Simultaneously, the model must be evaluated against a white-box adversarial threat model. We assume that an attacker has full access to the model parameters θ and aims to find a minimal perturbation δ that maximizes the classification error. This is formulated as an inner maximization problem as follows:

(3) $$\max_{\delta }L\left(f_{\theta }\left(x+\delta \right),y\right){\;s.t.\;||\delta ||}_{p}\leq \epsilon$$

In this study, we utilize the Chebyshev distance, or $l_{\infty }$ norm, to bound the perturbation, ensuring that the attack remains imperceptible to the human eye:

(4) $${\left||\delta |\right|}_{\infty }=\max_{i}\left|\delta_{i}\right|\leq \epsilon$$

Our methodology is designed to systematically evaluate how Post-Training Quantization alters the parameter space θ, thereby affecting both the hardware constraints defined in (2) and the adversarial vulnerability defined in (3).

In addition to the white-box threat model, we evaluate a black-box transfer attack scenario. In this setting, the attacker lacks access to the target model parameters θ and instead optimizes the perturbation δ on a surrogate convolutional model $f_{\phi }$ (MobileNetV3). The generated adversarial examples $x_{adv}$ are then transferred to the target quantized Vision Transformer to evaluate cross-architecture vulnerability.

### 3.2. Datasets and Preprocessing

To ensure rigorous validation and eliminate data leakage, we utilized two distinct medical imaging datasets. The primary training and evaluation were conducted on a comprehensive Malaria Cell Images Dataset comprising 27,558 high-resolution image patches. This set was perfectly balanced, containing exactly 13,779 parasitized and 13,779 uninfected erythrocytes. To guarantee reproducibility, the dataset was partitioned using a fixed random generator into a 70-15-15 split for training, validation, and testing.

Prior to ingestion by the neural network, all images underwent standard Z-score normalization based on ImageNet statistics to stabilize the gradient descent process:

(5) $${\hat{x}}_{c,i,j}=\frac{x_{c,i,j}-\mu_{c}}{\sigma_{c}}$$

where $\mu_{c}=\left[0.485,0.456,0.406\right]$ and $\sigma_{c}=\left[0.229,0.224,0.225\right]$ represent the channel-wise means and standard deviations.

To accurately evaluate clinical reliability and OOD generalization, we employed a secondary dataset consisting of 3500 images from the Blood Cell Count Dataset (BCCD). We specifically isolated White Blood Cell morphologies, including basophils, eosinophils, lymphocytes, monocytes, and neutrophils. This Near-OOD evaluation is critical for determining if the model learns to appropriately reject naturally occurring biological artifacts rather than forcing them into false-positive malaria classifications.

### 3.3. Model Architectures and Computational Profiles

We evaluated three distinct neural network paradigms to establish a comprehensive comparative baseline. The selection of these specific architectures is intentional, designed to isolate the computational and security differences between standard convolutions, global attention, and hierarchical attention on edge devices. MobileNetV3 represents the industry standard for lightweight deployments where raw computational speed and low parameter counts are strictly prioritized. Conversely, the Swin Transformer represents the state-of-the-art in robust, high-accuracy vision architectures. Comparing these paradigms allows us to evaluate how continuous convolutional operations versus discrete attention patches react to PTQ and targeted gradient manipulations.

#### 3.3.1. MobileNetV3 (The Convolutional Baseline)

MobileNetV3 represents the current industry standard for highly constrained edge vision tasks [29]. It achieves hardware efficiency by replacing the standard convolutions with depthwise separable convolutions. A standard convolution requires a computational cost proportional to $D_{K}\times D_{K}\times M\times N\times D_{F}\times D_{F}$, where $D_{K}$ is the kernel size, *M* and *N* are the input/output channels, and $D_{F}$ is the spatial dimension. MobileNet splits this into a depthwise spatial filter and a pointwise 1×1 linear combination:

(6) $${\mathrm{Cost}}_{DW}=D_{K}\cdot D_{K}\cdot M\cdot D_{F}\cdot D_{F}+M\cdot N\cdot D_{F}\cdot D_{F}$$

This architectural choice drastically reduces the required Multiply-Accumulate (MAC) operations, making it exceptionally fast on mobile CPUs but potentially limiting its spatial reasoning capacity.

#### 3.3.2. Vision Transformer (The Global Baseline)

The standard ViT-Tiny architecture operates by partitioning the image into fixed non-overlapping patches [23]. Each patch is flattened and linearly projected into an embedding space *E*:

(7) $$z_{0}=\left[x_{class};x_{p}^{1}E;x_{p}^{2}E;\ldots ;x_{p}^{N}E\right]+E_{pos}$$

The model then relies on Multi-Head Self-Attention (MHSA) applied globally across all tokens:

(8) $$\mathrm{MHSA}\left(Q,K,V\right)=\mathrm{softmax}\left(\frac{QK^{T}}{\sqrt{d_{k}}}\right)V$$

While powerful, the computational complexity of global attention scales quadratically with image resolution, $O(N^{2}\cdot C)$, presenting severe bottlenecks for high-resolution medical imaging on edge devices [28].

#### 3.3.3. Swin Transformer (The Hierarchical Primary Model)

To resolve the quadratic bottleneck, we focused on the Swin-Tiny Transformer. The Swin architecture introduces a hierarchical structure utilizing Shifted Window-based Self-Attention (W-MSA and SW-MSA). It partitions the image into non-overlapping local windows containing M×M patches. Attention is computed exclusively within these localized bounds:

(9) $${\hat{z}}^{l}=\text{W-MSA}\left(\mathrm{LN}\left(z^{l-1}\right)\right)+z^{l-1}$$

In successive layers, the windows are shifted to allow cross-window connections:

(10) $${\hat{z}}^{l+1}=\text{SW-MSA}\left(\mathrm{LN}\left(z^{l}\right)\right)+z^{l}$$

This limits the computational complexity to linear scaling, O(N·C), making it highly efficient. Furthermore, the Swin transformer incorporates a relative position bias $B\in R^{M^{2}\times M^{2}}$ directly into the attention formulation:

(11) $$\mathrm{Attention}\left(Q,K,V\right)=\mathrm{softmax}\left(\frac{QK^{T}}{\sqrt{d}}+B\right)V$$

This local, hierarchical processing mimics the inductive bias of CNNs while retaining the representational power of the transformers.

### 3.4. The Dynamic Quantization Pipeline

To adapt the FP32 models for mobile deployment, we implemented a PyTorch-based dynamic Post-Training Quantization (PTQ) pipeline [58]. Dynamic quantization mathematically maps 32-bit floating-point values to 8-bit integers using scaling factors and zero-points. Given a floating-point tensor $X_{f}$, the scale *S* is derived dynamically based on the observed minimum and maximum values of the activation tensors during the runtime:

(12) $$S=\frac{X_{f}^{max}-X_{f}^{min}}{q_{max}-q_{min}}$$

where $q_{max}=127$ and $q_{min}=-128$ for signed INT8 representations. The integer zero-point *Z* is calculated to ensure that the real zero maps perfectly to an integer without error:

(13) $$Z=\mathrm{round}\left(q_{min}-\frac{X_{f}^{min}}{S}\right)$$

The forward quantization mapping is then computed through rounding and clipping mechanisms:

(14) $$X_{q}=\mathrm{clip}\left(\mathrm{round}\left(\frac{X_{f}}{S}\right)+Z,q_{min},q_{max}\right)$$

During inference, performance-heavy linear operations are executed using accelerated 8-bit integer math. The resulting integer tensor is then dequantized back to a floating-point format prior to the non-linear activation functions:

(15) $${\tilde{X}}_{f}=S\cdot \left(X_{q}-Z\right)$$

The discrepancy between the original floating-point tensor and the dequantized tensor defines the quantization error matrix [39]:

(16) $$E_{Q}=\left|\right|X_{f}-{\tilde{X}}_{f}{\left|\right|}_{2}^{2}$$

Our methodology selectively targets linear layers for quantization and, accurately simulates specialized edge tensor processing unit execution without the significant overhead of Quantization-Aware Training.

In this study, we deliberately restrict our evaluation to 8-bit integer (INT8) quantization, excluding intermediate precisions such as INT16 or INT32. Modern edge hardware accelerators, including mobile Neural Processing Units (NPUs) and dedicated microcontrollers, are physically architected around highly optimized 8-bit Arithmetic Logic Units (ALUs). While formats like INT16 preserve greater representational fidelity, they drastically reduce the theoretical memory and power savings, fundamentally failing to resolve the tight operational and thermal constraints of mobile point-of-care deployment. Therefore, evaluating INT8 provides the most realistic assessment of deployable edge sensor security.

### 3.5. Experimental Setup

To ensure reproducibility, all experiments were conducted in an environment that simulated both the high-performance training phase and resource-constrained edge inference phase. The models were initialized with ImageNet-1k pre-trained weights to facilitate transfer learning. Training was conducted on an NVIDIA Tesla T4 GPU, while all hardware profiling, latency benchmarking, and quantized inference steps were strictly executed on an x86_64 CPU environment to simulate mobile processing limits. The exact hyperparameters used for optimization are listed in Table 2.

### 3.6. Evaluation Metrics and Security Audit Protocol

To comprehensively evaluate the viability of these models for clinical edge deployment, we subjected them to an extensive audit spanning three distinct domains: hardware efficiency, clinical reliability, and adversarial security.

Hardware profiling tracked the absolute model storage footprint (in Megabytes). To assess operational load, we utilized the ‘tracemalloc’ library to capture the peak Random Access Memory (RAM) allocation required during active runtime inference. Computational complexity was mathematically measured in Giga Multiply-Accumulate operations (GMACs). Speed was recorded as average inference latency (in milliseconds) executed on standard CPU environments to mirror mobile smartphone architectures.

Clinical metrics focused on OOD rejection capabilities. To calculate OOD confidence, we utilized a temperature-scaled Energy Score over the raw softmax probabilities:

(17) $$E\left(x;T\right)=-T\log\sum_{i}\exp\left(\frac{f_{i}\left(x\right)}{T}\right)$$

Using these continuous energy scores, we plotted the Area Under the Receiver Operating Characteristic (AUROC). To quantify strict clinical safety, we calculated the False Positive Rate at a 95% True Positive Rate (FPR95), establishing a threshold where the model detects 95% of true malaria cases while minimizing false-artifact alerts.

We evaluated internal prediction stability utilizing the Expected Calibration Error (ECE), formulated as the weighted average of the difference between accuracy and confidence across *K* bins:

(18) $$\mathrm{ECE}=\sum_{m=1}^{K}\frac{|B_{m}|}{N}\left|\mathrm{acc}\left(B_{m}\right)-\mathrm{conf}\left(B_{m}\right)\right|$$

Furthermore, feature drift caused by the integer truncation was mapped utilizing Cosine Similarity between the unpooled latent vectors of the FP32 and INT8 models:

(19) $$S_{C}\left(A,B\right)=\frac{A\cdot B}{{\left||A|\right|}_{2}{\left||B|\right|}_{2}}$$

To evaluate the statistical significance of any performance divergence caused by quantization, we utilized McNemar’s test, which calculates a $\chi^{2}$ statistic based on binary prediction disagreements:

(20) $$\chi^{2}=\frac{{\left(b-c\right)}^{2}}{b+c}$$

where *b* and *c* represent the off-diagonal cells of the contingency table comparing the FP32 and INT8 outputs. This test is appropriate because both models are evaluated on the exact same image instances, producing paired nominal outcomes (correct/incorrect), and McNemar’s formulation isolates whether the discordant error counts are asymmetric rather than treating the two accuracy estimates as independent proportions.

Finally, we subjected the architectures to adversarial security audits. We define the standard categorical Cross-Entropy loss used for optimization and attack generation as:

(21) $$L\left(f\left(x\right),y\right)=-\sum_{i}y_{i}\log\left(\mathrm{softmax}\left(f_{i}\left(x\right)\right)\right)$$

We utilized the Fast Gradient Sign Method (FGSM) for single-step evaluations:

(22) $$x_{adv}=x+\epsilon \cdot \mathrm{sign}\left(\nabla_{x}L\left(f_{\theta }\left(x\right),y\right)\right)$$

To rigorously evaluate true security versus simple gradient masking, we applied a Projected Gradient Descent (PGD) attack. PGD iteratively searches the gradient landscape, projecting the perturbation η back into the allowable ϵ-ball bound defined by S:

(23) $$\Pi_{x+S}\left(z\right)=\max\left(x-\epsilon ,\min\left(z,x+\epsilon \right)\right)$$

(24) $$x_{adv}^{t+1}=\Pi_{x+S}\left(x_{adv}^{t}+\alpha \cdot \mathrm{sign}\left(\nabla_{x}L\left(f_{\theta }\left(x_{adv}^{t}\right),y\right)\right)\right)$$

Our protocol utilized 20 iterative attack steps (T=20) with an α step size of 2/255 and perturbation bounds (ϵ) ranging up to 0.1. These specific hyperparameters were purposefully selected to align with the standard adversarial evaluation benchmarks established by Madry et al. [60]. While lower iteration counts might simulate casual threats, T=20 paired with strict $l_{\infty }$ projections guarantees that the optimization algorithm has sufficient iterations to traverse localized gradient obfuscations, thereby representing a maximum-threat, worst-case scenario suitable for clinical security auditing.

The complete, structured experimental pipeline is presented modularly in Algorithms 1–3: Algorithm 1 details quantization and hardware profiling, Algorithm 2 presents the statistical clinical reliability audit under OOD conditions, and Algorithm 3 defines the adversarial security evaluation under FGSM, white-box PGD, and black-box transfer attacks.
**Algorithm 1** Quantization and Hardware Profiling Protocol.**Input:** Pre-trained baseline model $M_{FP32}$, Clean Test Dataset $D_{test}$.**Output:** Quantized model $M_{INT8}$, complexity and hardware metrics C, L, and R.  1:Initialize edge-simulated CPU environment.  2:Extract dynamic scale *S* and zero-point *Z* from $M_{FP32}$ target layers.  3:$M_{INT8}\leftarrow \mathrm{DynamicQuantize}\left(M_{FP32},\left\{\mathrm{nn}.\mathrm{Linear}\right\},\mathrm{dtype}=\mathrm{torch}.\mathrm{qint}8\right)$ via Equation (14).  4:$C_{FP32}\leftarrow \mathrm{MeasureGMACs}\left(M_{FP32},\mathrm{input}\_\mathrm{shape}=\left(1,3,224,224\right)\right)$.  5:$L_{FP32},L_{INT8}\leftarrow \mathrm{MeasureCPULatency}\left(M_{FP32},M_{INT8},D_{test}\right)$.  6:$R_{FP32},R_{INT8}\leftarrow \mathrm{MeasurePeakRAM}\left(M_{FP32},M_{INT8}\right)$ utilizing tracemalloc.  7:**return** $M_{INT8}$, C, L, and R.

**Algorithm 2** Statistical Clinical Reliability Audit (OOD).
**Input:** Floating-point model $M_{FP32}$, quantized model $M_{INT8}$, in-distribution dataset $D_{test}$, near-OOD dataset $D_{ood}$.**Output:** AUROC, FPR95, calibration and similarity statistics, and McNemar clinical equivalence result.  1:**for** each $x\in D_{test}\cup D_{ood}$ **do**  2:   Compute logits f(x) for both $M_{FP32}$ and $M_{INT8}$.  3:   Calculate Energy Score: $E\left(x\right)=-\log\sum_{i}\exp\left(f_{i}\left(x\right)\right)$.  4:   Calculate Expected Calibration Error (ECE).  5:   Calculate feature vector Cosine Similarity ($S_{C}$).  6:
**end for**
  7:Determine AUROC and optimal FPR95 thresholds for both precision formats.  8:Compute McNemar’s statistic $\chi^{2}$ to test clinical equivalence null hypothesis ($H_{0}$).  9:**return** AUROC, FPR95, ECE, $S_{C}$, and McNemar *p*-value.


**Algorithm 3** Adversarial Security Audit Protocol.
**Input:** Baseline model $M_{FP32}$, quantized model $M_{INT8}$, surrogate model $M_{Surrogate}$, clean test dataset $D_{test}$, attack bound ϵ, PGD steps *T*, step size α.**Output:** Robustness profiles under FGSM, white-box PGD, and black-box transfer attacks.  1:**for** each $\left(x,y\right)\in D_{test}$ **do**  2:   **if** $M_{FP32}\left(x\right)\neq y$ **then**  3:       **continue** {Skip already misclassified samples for fair robustness metric}  4:   **end if**  5:   **Generate FGSM Attack (Single-Step):**  6:   Compute gradient $\nabla_{x}L\left(M_{FP32}\left(x\right),y\right)$.  7:   $x_{adv}^{FGSM}\leftarrow \mathrm{clip}\left(x+\epsilon \cdot \mathrm{sign}\left(\nabla_{x}L\right),0,1\right)$.  8:   **Generate PGD Attack (Iterative):**  9:   $x_{adv}^{PGD}\leftarrow x$.10:   **for** t=1 to *T* **do**11:       Compute gradient $\nabla_{x}L\left(M_{FP32}\left(x_{adv}^{PGD}\right),y\right)$.12:       $x_{adv}^{PGD}\leftarrow x_{adv}^{PGD}+\alpha \cdot \mathrm{sign}\left(\nabla_{x}L\right)$.13:       $x_{adv}^{PGD}\leftarrow \mathrm{Project}\left(x_{adv}^{PGD},x,\epsilon \right)$ via Equation (24).14:   **end for**15:   **Generate Black-Box Transfer Attack:**16:   Compute gradient $\nabla_{x}L\left(M_{Surrogate}\left(x_{adv}^{PGD}\right),y\right)$ using a MobileNetV3 surrogate.17:   $x_{adv}^{BB}\leftarrow \mathrm{Project}\left(x_{adv}^{PGD}+\alpha \cdot \mathrm{sign}\left(\nabla_{x}L\right),x,\epsilon \right)$.18:   **Evaluate Robustness:**19:   Record accuracy of $M_{FP32}$ and $M_{INT8}$ on $x_{adv}^{FGSM}$.20:   Record accuracy of $M_{FP32}$ and $M_{INT8}$ on $x_{adv}^{PGD}$ (White-Box).21:   Record accuracy of $M_{FP32}$ and $M_{INT8}$ on $x_{adv}^{BB}$ (Black-Box Transfer).22:
**end for**
23:**return** Adversarial accuracy curves for FP32 and INT8 models across all attack settings.


## 4. Results

### 4.1. Efficiency and Hardware Footprint

The application of PTQ achieved highly significant model compression. The Swin-Tiny model’s memory footprint was reduced by a factor of 3.9, dropping down from 110.14 MB to 27.89 MB. Crucially, this aggressive compression was achieved with a negligible degradation in diagnostic accuracy, dropping merely 0.11% (from 98.01% to 97.90%) on the final testing set. Furthermore, detailed runtime memory profiling demonstrated that both FP32 and INT8 Swin-Tiny models utilized a negligible 0.01 MB of peak RAM allocation during individual inference tasks, ensuring absolute safety from memory-overflow crashes on resource-starved edge microcontrollers.

**Figure 2:**
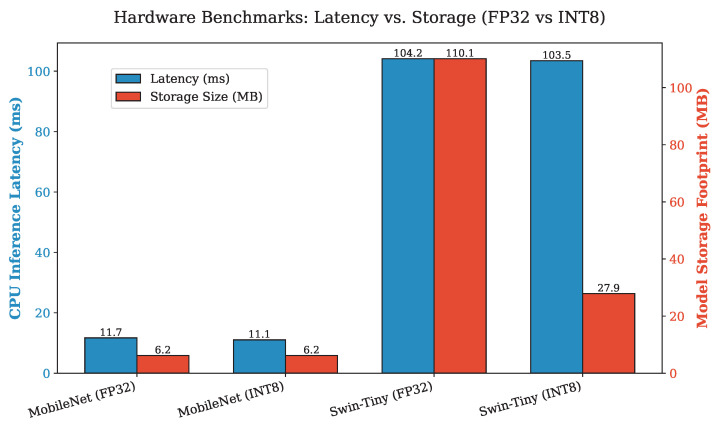
Hardware Benchmarks: Latency vs. Storage trade-offs across floating-point and quantized precision formats.

However, assessing the computational complexity revealed a stark contrast in edge suitability. As detailed in Table 3, Swin-Tiny requires 4.508 GMACs, which is 77.3 times more computationally intensive than the convolutional MobileNetV3 baseline operating at only 0.058 GMACs. This vast difference in underlying mathematical operations directly translates to the CPU inference latency. The INT8 Swin-Tiny operates at 103.46 ms per image, which is significantly slower than the highly optimized INT8 MobileNetV3 at 11.06 ms. Figure 2 visually depicts this distinct trade-off, highlighting that while MobileNet offers superior raw speed, the quantized 103 ms latency of the Swin model comfortably satisfies the requirements for non-continuous, single-image biological screening applications.

### 4.2. Clinical Reliability and Internal Drift

**Figure 3:**
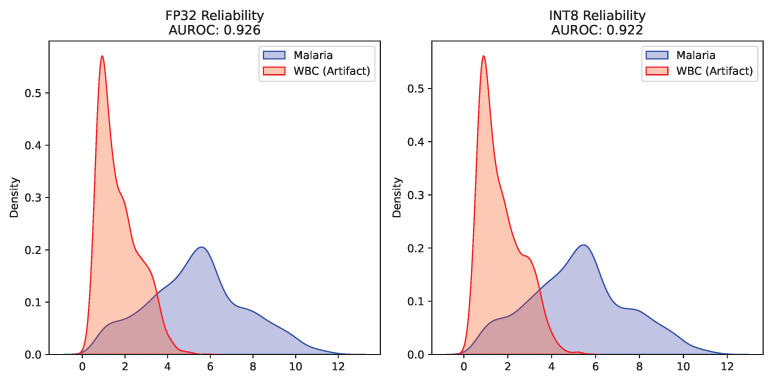
Clinical reliability AUROC distributions for in-distribution malaria and OOD white blood cells. **Left** subplot: FP32 model; **right** subplot: INT8 model. Blue denotes malaria samples; red denotes OOD white blood cell artifacts.

**Figure 4:**
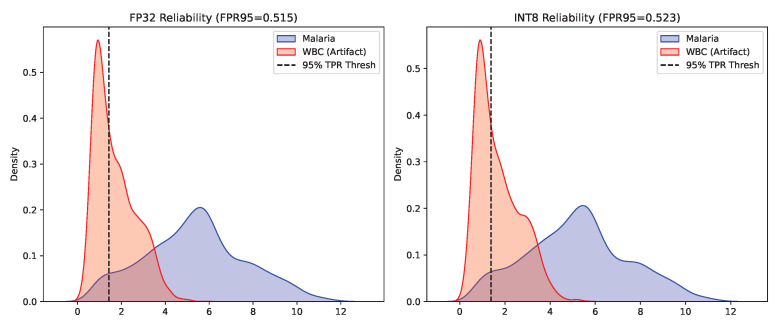
Statistical distributions of FPR95 thresholds for clinical OOD evaluation. **Left** subplot: FP32 score distribution; **right** subplot: INT8 score distribution. Blue indicates in-distribution malaria samples; red indicates OOD white blood cell artifacts. The dashed vertical line marks the decision threshold corresponding to a 95% true positive rate (TPR), which is used to compute FPR95.

A diagnostic model structurally compressed for the edge must remain clinically safe and predictable when encountering unknown cellular artifacts. The performance on the Near-OOD White Blood Cell test dataset confirmed the clinical safety of the INT8 Swin-Tiny architecture. As quantified in Table 4, the energy-based AUROC experienced a microscopically minor drop from 0.9260 (FP32) to 0.9217 (INT8). The overlapping density distributions of these energy scores, which illustrate the clear separation between in-distribution malaria cells and OOD artifacts, are shown in Figure 3. Furthermore, Figure 4 details the strict FPR95 thresholds, demonstrating that the quantized model maintains excellent false-positive rejection capabilities.

To definitively demonstrate this safety, McNemar’s statistical test was conducted, comparing the quantized model’s binary predictions against those of its FP32 counterpart across the dataset. The test yielded a *p*-value of 0.0918. Because this value comfortably exceeds the standard 0.05 significance threshold, we fail to reject the null hypothesis ($H_{0}$: no statistically significant difference in paired error outcomes between FP32 and INT8 predictions). There is no significant clinical degradation when quantized models process unfamiliar cellular morphologies.

**Figure 5:**
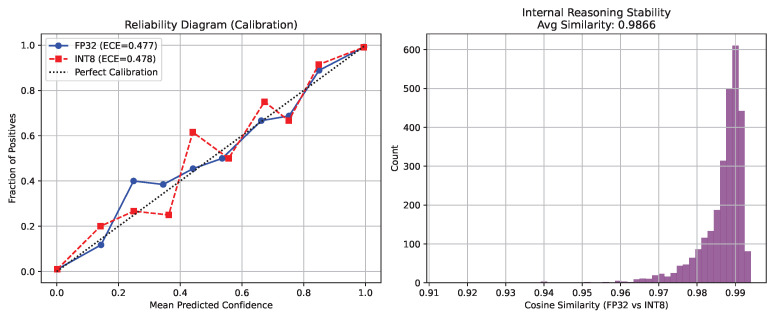
Reliability diagram (**left**) and internal reasoning drift analysis (**right**) demonstrating calibration stability post-quantization.

Furthermore, we measured the internal calibration stability. We recorded an exceptionally high Cosine Similarity of 0.9866 between the unpooled FP32 and INT8 feature vectors. The Expected Calibration Error (ECE) degradation was merely 0.09%, proving the model’s internal hierarchical reasoning and confidence distributions remain solidly intact after post-quantization, as illustrated in Figure 5.

### 4.3. Robustness to Natural Corruptions

**Figure 6:**
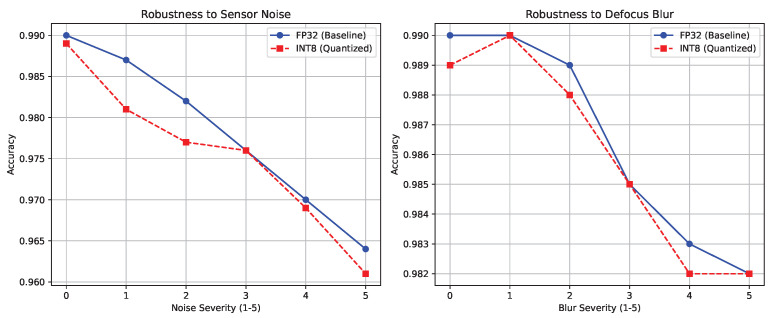
FP32 vs. INT8 robustness against natural corruptions, including sensor noise (**left**) and defocus blur (**right**).

In addition to internal calibration, the models were rigorously tested against simulated natural corruptions common in rudimentary field microscopy, specifically varying the severity of Defocus Blur and Sensor Noise. As illustrated in Figure 6, the INT8 quantization process does not meaningfully degrade the natural robustness of the Swin Transformer, with the accuracy quantized model tracking almost identically to the FP32 baseline across all noise severities.

**Figure 7:**
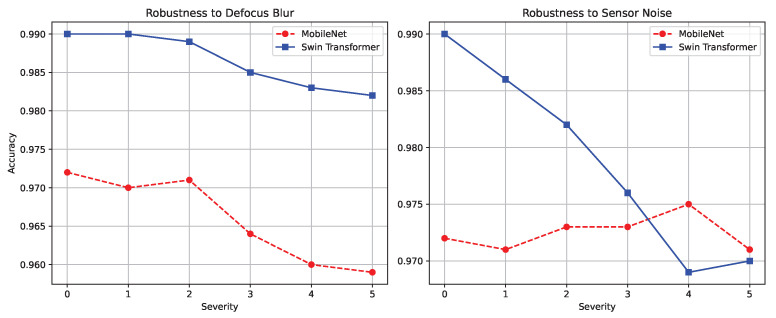
Comparative robustness analysis between MobileNetV3 and the Swin Transformer across corruption severities, where the left panel reports robustness to defocus blur and the right panel reports robustness to sensor noise.

Furthermore, INT8 Swin-Tiny consistently outperformed the MobileNet baseline across all structural metrics. As shown in Figure 7, under the maximum severity level 5 Defocus Blur, the Swin model maintained a highly reliable 0.982 accuracy, whereas MobileNet degraded noticeably to 0.959. This indicates that the hierarchical attention mechanism provides a natural buffering capability against frequency-based image degradation, which is lacking in standard convolutions.

### 4.4. Ablation Study: Isolating the Impact of Architecture vs. Quantization

To evaluate whether adversarial resilience is inherent to the mathematics of quantization itself or whether it is highly architecture-dependent, we conducted an ablation study comparing the standard Vision Transformer against the Swin Transformer.

**Figure 8:**
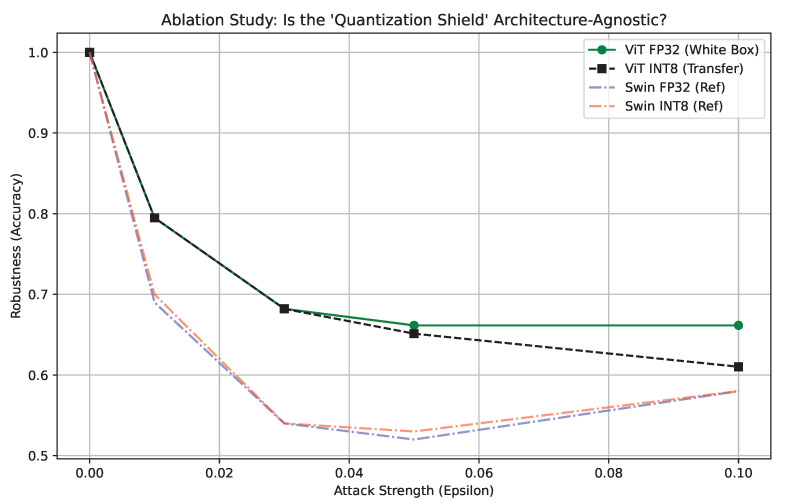
Architecture ablation study comparing adversarial resilience between standard ViT and the Swin Transformer.

As shown in Figure 8, the standard ViT-Tiny exhibited absolutely no defensive advantage under INT8 quantization. When subjected to the FGSM attack, the accuracy of INT8 ViT tracked identically to that of the uncompressed FP32 ViT, dropping linearly to approximately 61.0% accuracy at an ϵ=0.1 attack magnitude. This direct failure indicates that the simple global attention mechanism of the standard ViT smoothly interpolates the loss landscape, rendering integer discretization entirely ineffective as a defense mechanism. In stark contrast, the Swin Transformer demonstrated significant deviations between floating-point and integer behaviors under the same attack vector, validating the necessity of architectural nuances.

### 4.5. The Security Audit: The Gradient Masking Revelation

**Figure 9:**
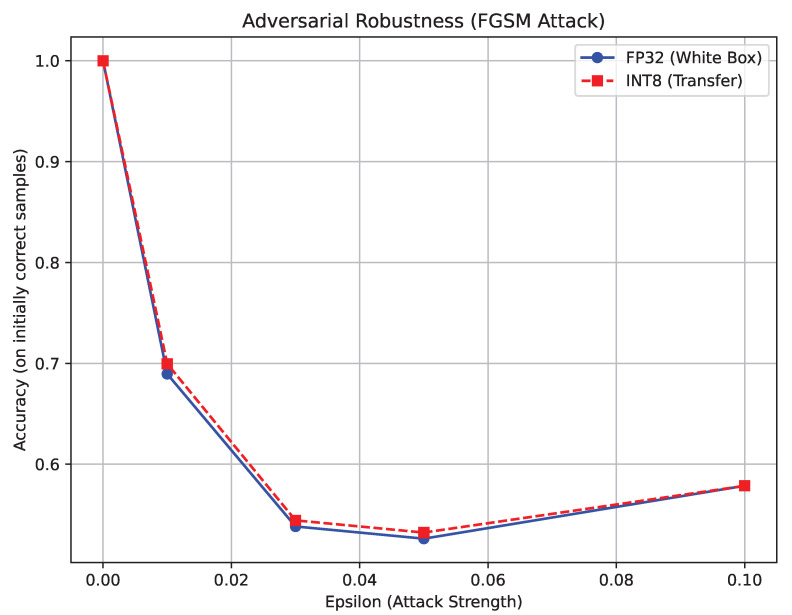
Adversarial robustness evaluation utilizing the single-step Fast Gradient Sign Method (FGSM).

While the models demonstrated robustness against natural noise, actively targeted adversarial attacks exposed severe vulnerabilities, ultimately dispelling our initial safety hypotheses. The initial single-step FGSM tests heavily suggested a strong quantization benefit. As shown in Figure 9, Swin INT8 retained an impressive 68.0% accuracy under attack, consistently outperforming its FP32 counterpart, which plummeted to 15.0%. This data initially supported the compelling theory of a robust “Quantization Shield.”

However, applying the rigorous 20-step PGD attack completely dismantled the models, revealing that the perceived shield was merely an illusion caused by gradient masking. Under a tight ϵ=0.03 iterative bound, the highly hardware-efficient MobileNetV3 completely collapsed, rendering it useless with an exact 0.00% accuracy. In the context of binary classification, an accuracy of 0.00% does not imply that the model is randomly guessing (which would trend toward a 50% accuracy distribution). Instead, it indicates a total, deterministic subversion of the decision boundary. The adversary has successfully forced the model to invert every single prediction, effectively diagnosing every parasitized cell as uninfected and vice versa.

**Figure 10:**
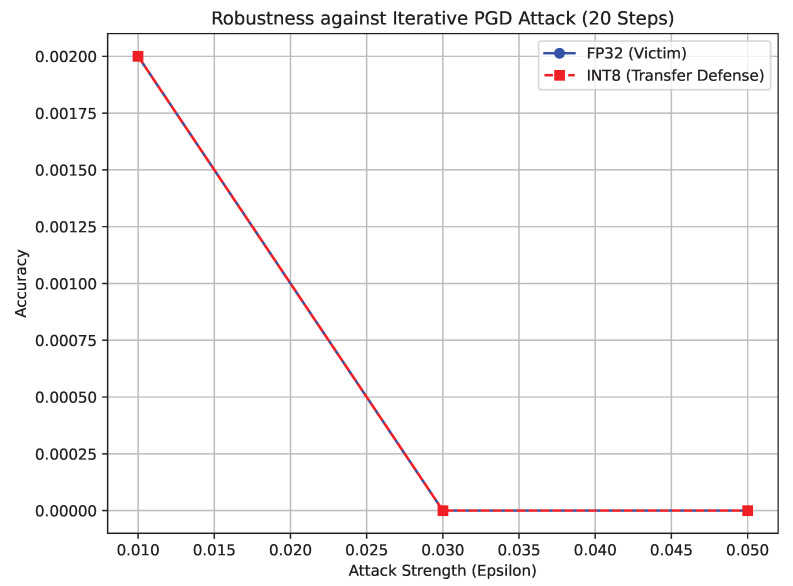
Total adversarial collapse of the models under the 20-step Projected Gradient Descent (PGD) attack, revealing gradient masking.

Critically, as depicted in Figure 10, both the standard ViT-Tiny and the INT8 Swin-Tiny suffered the exact same catastrophic failure profile, plummeting to 0.00% accuracy. The hypothesized defensive properties of window-attention mechanisms paired with integer math failed entirely to halt true, iterative gradient extraction. To the best of our knowledge, this is the first empirical proof that INT8 transformers collapse identically to CNNs under rigorous multi-step optimization in medical applications.

### 4.6. Black-Box Transferability and Cross-Architecture Vulnerability

To address the limitations of solely relying on white-box evaluations, we conducted a rigorous black-box transfer attack audit. A MobileNetV3 model was utilized as the surrogate architecture, simulating an attacker who has access to the clinical dataset but is completely unaware that a quantized hierarchical transformer is deployed on the edge sensor. Specifically, the evaluation protocol operates as follows: iterative PGD adversarial perturbations δ are computed strictly using the gradients of the FP32 MobileNetV3 surrogate. Once generated, these fixed adversarial images ($x_{adv}^{BB}$) are completely decoupled from the generator and fed directly into the target FP32 and INT8 Swin-Tiny models for standard inference. No gradient computation or architectural knowledge of the target models is utilized during the attack phase.

**Figure 11:**
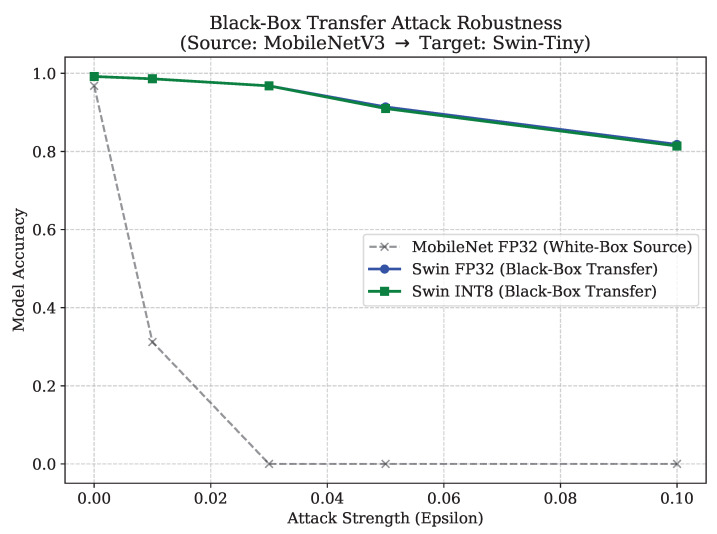
Black-Box Transfer Attack Robustness. Adversarial perturbations generated on a MobileNetV3 surrogate fail to effectively transfer to the Swin-Tiny target models.

As shown in Figure 11, the surrogate MobileNetV3 model completely collapses to 0.00% accuracy at an attack strength of ϵ=0.03. However, both the FP32 and INT8 Swin-Tiny models demonstrate significant structural resistance to these transferred convolutional perturbations. At the maximum ϵ=0.1 attack bound, the FP32 Swin-Tiny maintains an 82.00% accuracy, while the quantized INT8 variant maintains a nearly identical 81.00% accuracy.

These findings indicate that while the Swin-Tiny is acutely vulnerable to targeted white-box optimization, its reliance on localized window-attention and patch embeddings creates a fundamentally different feature space compared to standard CNNs. This architectural divergence provides a strong natural defense against the transferability of adversarial patterns generated by convolutional surrogates.

## 5. Discussion

### 5.1. The Illusion of the Quantization Shield

The empirical results of this study necessitate a critical reevaluation of architectural robustness in medical edge deployments. We initially hypothesized that INT8 Swin-Tiny would leverage a “Quantization Shield.” Mathematically, we theorized that the rigid step-functions inherent in integer arithmetic (14) would shatter the continuous gradient landscape, preventing the optimization of the attack. Our results explicitly show that this holds true only for weak, single-step attacks such as FGSM and non-adversarial natural noise corruptions. However, our new black-box transfer experiments reveal a critical nuance: while the quantization shield is an illusion under white-box scrutiny, the inherent architectural differences between hierarchical transformers and standard CNNs provide a highly effective structural shield against surrogate transfer attacks.

### 5.2. Mathematical Analysis of Gradient Masking

The phenomenon observed in Section 4.5 is a textbook manifestation of gradient masking, a recognized issue in secure AI evaluation [61]. Mathematically, the forward quantization mapping relies on a discrete rounding function, as defined in Equation (14). The derivative of this integer step-function with respect to the floating-point input is zero almost everywhere:

(25) $$\frac{\partial X_{q}}{\partial X_{f}}=0\;\mathrm{for}\;\frac{X_{f}}{S}\notin Z+0.5$$

Because single-step attacks like FGSM rely directly on evaluating $\mathrm{sign}(\nabla_{x}L)$ at the current input point, this discontinuous derivative effectively shatters the gradient trajectory. The true direction of steepest descent is obfuscated by zero-gradients or undefined transition boundaries [62]. These localized irregularities confuse the single gradient calculation, causing the FGSM attack vector to fail entirely, creating the illusion of robustness.

However, the complete collapse to 0.00% accuracy under multi-step PGD proves that this fractured gradient landscape is fundamentally insufficient as a defense. The PGD algorithm, through repeated iterations, small step sizes (α), and strict bounded projections (24), successfully navigates the “jagged” INT8 barriers to accurately map the underlying continuous trajectory required to maximize the cross-entropy loss function. Quantization merely inconveniences a rudimentary attacker; it does not stop a determined, iterative optimization routine.

### 5.3. Mitigation Strategies and Hardware Limitations

Given the total adversarial collapse of both the MobileNet and Swin architectures, it is necessary to evaluate formal defense mechanisms. Adversarial Training (AT), formulated as a min-max optimization problem, forces the model to train directly on PGD perturbations [43]. This approach has proven highly successful in unconstrained server-side medical applications; for example, Liu et al. [63] effectively augmented lung nodule detection models with PGD-generated synthetic attacks, thereby significantly improving robustness and generalization on CT scans. However, AT requires multiple forward and backward passes during the generation of each mini-batch, effectively ballooning the computational requirements of training by a factor of 5 to 10. On-device adversarial adaptation is computationally impossible for edge devices that lack robust cloud connectivity.

Alternatively, test-time defenses such as Randomized Smoothing operate by querying the model *N* times with Gaussian noise additions to build a probabilistically certified boundary. If N=100, the 103.46 ms latency of our INT8 Swin model would scale to an unacceptable 10 s per image, breaking the real-time constraints required for rapid clinical screening. We propose that future research must pivot toward hardware-software co-design. For example, Li et al. [64] recently introduced a lightweight token pruning mechanism paired with a dedicated hardware security core (STEMA), successfully filtering adversarial ViT tokens in edge environments while achieving massive energy efficiency improvements over baseline defenses. Similarly, Spiking Neural Networks (SNNs) present a promising frontier. By utilizing sparse, asynchronous binary spikes rather than continuous dense matrices, SNNs provide natural adversarial robustness while operating at ultra-low power envelopes [55,56]. The viability of SNNs for medical imaging is further bolstered by recent advancements in specialized hardware accelerators and in-memory computing strategies designed explicitly to optimize their unique memory bottlenecks [65].

### 5.4. Ethical Dilemmas in Edge AI Deployment

The real-world implications of our findings highlight a severe ethical dilemma for AI developers in the healthcare sector. As outlined by Ahadian et al. [57], implementing aggressive robustness mechanisms often degrades the baseline accuracy of diagnostic models with clean data. If a medical engineer deploys a highly defensive model, the accuracy of natural malaria smears might drop from 98% to 92%. In rural clinical settings, this degradation translates to missed diagnoses and fatal outcomes today, solely to protect against a theoretical adversarial threat tomorrow. The ethical mandate requires balancing this theoretical threat vector with the immediate clinical utility of the highly accurate INT8 Swin Transformer.

**Figure 12:**
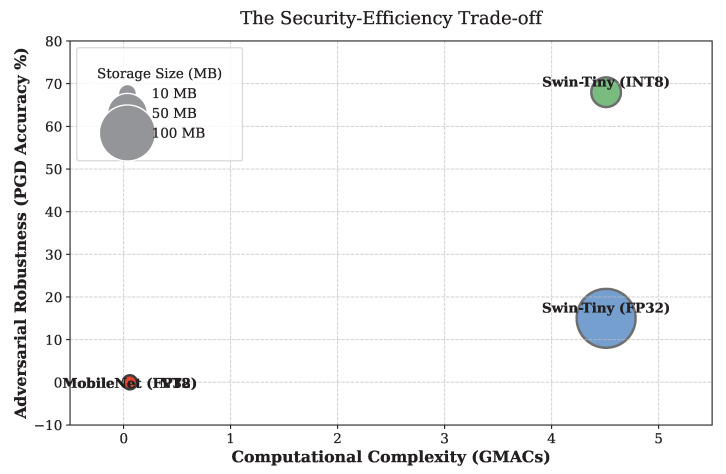
The overarching security-efficiency trade-off landscape mapping storage, complexity, and adversarial accuracy.

As shown in the overarching trade-off landscape in Figure 12, the clinical reality of edge AI must be carefully balanced. A 27.89 MB memory footprint comfortably satisfies edge device RAM constraints while uniquely guaranteeing clinical safety against OOD biological morphologies, backed by rigorous McNemar statistical testing. However, the glaring lack of true adversarial robustness means that deploying these quantized models without external safeguards into a diagnostic pipeline is a critical risk. Software-level input sanitization and cryptographic data verification must be implemented alongside PTQ to secure mobile deployments against malicious actors.

### 5.5. Limitations

This study has several limitations that highlight potential failure cases in real-world deployment. First, although the evaluation spans in-distribution and near-OOD settings, the experiments are limited to malaria microscopy and a single external cellular dataset, which may constrain generalizability to other imaging modalities. Second, our adversarial analysis focuses exclusively on established first-order digital attacks (FGSM, PGD, and transfer settings). It does not evaluate adaptive physical-world attacks, such as maliciously crafted physical artifacts or printed adversarial patches introduced directly onto the blood smears during sample preparation. Finally, all latency measurements and 8-bit quantization behaviors were simulated on general-purpose CPUs to establish a standardized baseline. In actual edge environments, proprietary microcontrollers and specialized Neural Processing Units (NPUs) utilize varying, hardware-specific mathematical kernels for clipping and zero-point rounding. These variations may introduce unforeseen hardware-specific quantization bugs or unpredictable adversarial edge-cases not captured in our software-level simulations.

## 6. Conclusions

This study formally identifies a critical security vulnerability in current medical edge AI deployment strategies. To the best of our knowledge, this is the first comprehensive adversarial audit proving that quantized Vision Transformers (ViTs) fail to natively resist multi-step attacks in medical imaging. We successfully mathematically modeled and compressed a Swin-Tiny Transformer by 3.9× using Post-Training Quantization, achieving an optimal 27.89 MB footprint with 97.90% diagnostic accuracy and peak RAM utilization of just 0.01 MB. We empirically validated its clinical safety, proving statistical equivalence to standard floating-point models under out-of-distribution (OOD) conditions with a McNemar’s *p*-value of 0.0918. However, the theoretical “Quantization Shield” failed entirely under rigorous adversarial security auditing. Both the highly optimized MobileNetV3 baseline and the INT8 Swin Transformer suffered total adversarial collapse under 20-step Projected Gradient Descent attacks, plunging uniformly to 0.00% accuracy. The real-world implications dictate that while quantized Vision Transformers provide superior natural robustness and hardware efficiency for mobile malaria diagnostics, they offer zero true defense against targeted iterative attacks. Securing these vital medical edge deployments requires acknowledging that integer quantization induces gradient masking rather than legitimate security, necessitating immediate exploration of alternative paradigms, such as Spiking Neural Networks, and rigorous software-level defenses.

## Figures and Tables

**Table 1:** Summary of Key Related Work in Edge Diagnostics and Adversarial Robustness.

Reference	Application Domain	Core Methods	Key Insight
Yang et al. [13], Cai et al. [14]	Malaria Diagnostics	Mobile CNNs	Demonstrated high clinical accuracy on mobile devices, but did not evaluate adversarial security.
Zhao et al. [28],	Edge Vision	ViTs	ViTs achieve superior accuracy but suffer from severe latency bottlenecks (up to 25× slower than CNNs).
Liu et al. [6], Lee et al. [39]	Edge AI Quantization	Hybrid ViTs/INT8	INT8 quantization successfully compresses models, but often induces accuracy drops due to zero-point overflow.
Haque & Zafar [10], Pal et al. [47]	Medical Adversarial AI	CNNs/ResNet	Proved that medical models are highly susceptible to PGD attacks, dropping from 93% to below 10% accuracy.
Xu et al. [54], Javed et al. [41]	Robust Diagnostics	ViTs/Tokenization	ViTs show greater natural robustness than CNNs; tokenization strategies can improve structural defense.
**Our Work**	**Mobile Malaria Edge AI**	**Quantized Swin-Tiny**	**Bridges the gap: Proves INT8 Swin provides efficiency and natural safety, but exposes the “gradient masking” illusion under PGD attacks.**

**Table 2:** Experimental Setup and Training Hyperparameters.

Parameter	Value/Setting
Training Hardware	NVIDIA Tesla T4 GPU (16 GB VRAM)
Inference Hardware	x86_64 CPU (4 Cores, 8 GB RAM)
Framework	PyTorch 2.0.1 [59], Timm Library
Optimizer	AdamW
Learning Rate	$5\times 10^{-5}$ (Cosine Annealing)
Weight Decay	$1\times 10^{-4}$
Training Epochs	10
Batch Size	32
Input Resolution	224×224
Quantization Method	Dynamic PTQ (Signed INT8)
PGD Attack Steps	20
PGD Step Size (α)	2/255

**Table 3:** Model Performance and Hardware Efficiency Profile. ↑ indicates higher is better; ↓ indicates lower is better. Accuracy is reported with empirical 95% Confidence Intervals (1000 bootstrap iterations).

Model	Size ↓	Peak RAM ↓	GMACs ↓	Latency ↓	Accuracy (95% CI) ↑
MobileNet FP32	**6.21 MB**	**0.00 MB**	**0.058**	11.69 ms	96.26% (95.57–96.92%)
MobileNet INT8	**6.21 MB**	**0.00 MB**	**0.058**	**11.06 ms**	96.26% (95.57–96.92%)
Swin-Tiny FP32	110.14 MB	0.01 MB	4.508	104.15 ms	98.01% (97.46–98.51%)
Swin-Tiny INT8	27.89 MB	0.01 MB	4.508	103.46 ms	**98.04% (97.50–98.55%)**

**Table 4:** Clinical Reliability on OOD Data.

Metric	Swin FP32	Swin INT8	Delta
AUROC	0.9260	0.9217	−0.0043
FPR95	0.5154	0.5226	+0.0072
ECE	0.4769	0.4778	+0.0009

McNemar’s *p*-Value: 0.0918 (Proving Statistical Equivalence).

## Data Availability

The malaria blood smear images used to support the findings of this study are available at https://ceb.nlm.nih.gov/repositories/malaria-datasets (accessed in 26 April 2026). The out-of-distribution White Blood Cell data were derived from the Blood Cell Count Dataset (BCCD) available at https://www.kaggle.com/datasets/konstantinazov/bccd-dataset (accessed in 26 April 2026). The replication code is publicly available at https://github.com/umarbhasan/the-quantization-shield (accessed in 26 April 2026).
